# Loss of CD22 expression and expansion of a CD22^dim^ subpopulation in adults with relapsed/refractory B-lymphoblastic leukaemia after treatment with Inotuzumab-Ozogamicin

**DOI:** 10.1007/s00277-021-04601-0

**Published:** 2021-07-31

**Authors:** Jochim Reinert, Antonia Beitzen-Heineke, Klaus Wethmar, Matthias Stelljes, Walter Fiedler, Stefan Schwartz

**Affiliations:** 1 Department of Oncology, Haematology and Bone Marrow Transplantation With Section Pneumology, Hubertus Wald Tumorzentrum, University Medical Center Hamburg-Eppendorf, Hamburg, Germany; 2 Department of Medicine A, Hematology and Oncology, University Hospital Münster, Münster, Germany; 3 Department of Haematology, Oncology and Cancer Immunology, Charité – Universitätsmedizin Berlin, corporate member of Freie Universität and Humboldt-Universität Zu Berlin, Campus Benjamin Franklin, Berlin, Germany

**Keywords:** B-ALL, CD22, Inotuzumab-Ozogamicin, Immunotherapy

## Abstract

Treatment options for relapsed or refractory B-lymphoblastic leukaemia (r/r B-ALL) are limited and the prognosis of these patients remains dismal, but novel immunotherapeutic options such as the anti-CD22 antibody–drug-conjugate Inotuzumab-Ozogamicin (InO) have improved outcomes in these patients. Flow cytometry is essential to assess antigen-expression prior to treatment initiation of antigen-directed immunotherapies. Here, we present flow cytometric and clinical data of three adult patients with r/r B-ALL who failed treatment with InO associated with reduced or lost antigen-expression. In addition, we present comparative data on two different diagnostic CD22-specific antibody clones that exhibit significant differences in staining intensities.

## Introduction

The prognosis of patients with refractory B-lymphoblastic leukaemia and those with early recurrence after chemotherapy (r/r B-ALL) is poor and treatment options are limited. Options for salvage-therapy include the anti-CD22 immunotherapeutic Inotuzumab-Ozogamicin (InO). This antibody–drug conjugate has shown superior rates of complete remissions (CR) and improved overall survival (OS) compared to intensive salvage-chemotherapy protocols in r/r B-ALL [1]. Furthermore, InO is a promising therapeutic option for combination treatments and is currently being tested in first-line treatment of newly diagnosed B-ALL [2]. Here, we present data from a series of three adult patients with r/r B-ALL, who failed treatment with InO associated with reduced CD22 antigen expression or who developed CD22-negative relapse as revealed by flow cytometry. With respect to a standardized flow cytometry procedure, we evaluated two different diagnostic CD22 antibody clones.

## Results

**Fig. 1:**
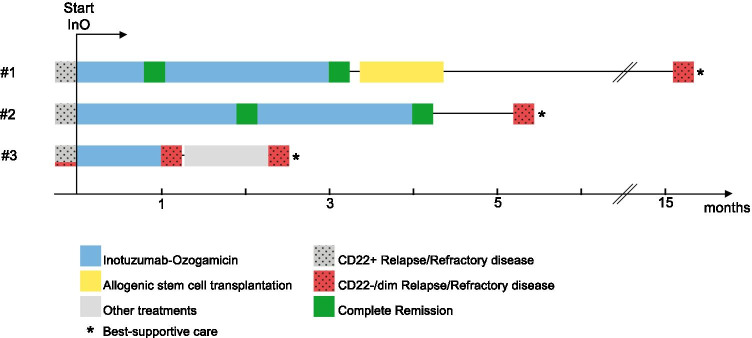
Overview of clinical course of the presented patients after initiation of treatment with Inotuzumab-Ozogamicin

Overall, data from 2 patients with primary refractory (patient 1, patient 3) and another patient with relapsed (patient 2) B-ALL were available. Two patients showed initial response to InO, but later developed CD22-negative relapse. In contrast, patient 3 failed salvage treatment with InO with emergence of a pre-existing CD22^dim^ subpopulation. Figure 1 provides an overview of treatment durations and responses after initiation of InO.

### Patient 1

**Fig. 2:**
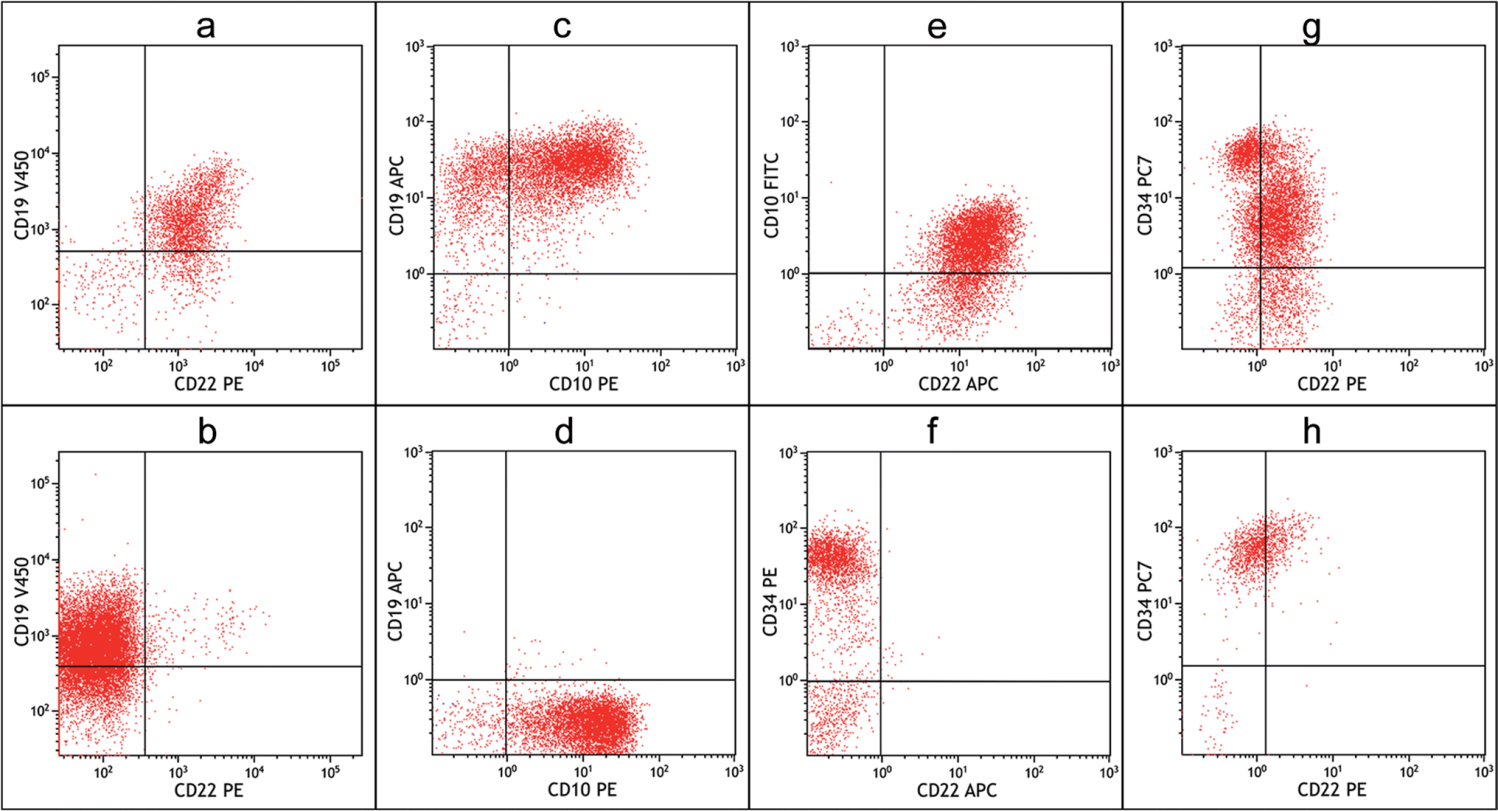
Flow cytometric evaluation of patient 1 (**a**, **b**), patient 2 (**c**–**f**) and patient 3 (**g**, **h**) for expression of target antigens before (top row) and after treatment (bottom row) with targeted immunotherapy. Patient 1 showed homogeneous CD22-positive blast population before treatment with InO (**a**) but presented with CD22-negative relapse 10 months after salvage-therapy with InO and allogenic haematopoietic stem cell transplantation (**b**). In patient 2, flow cytometry revealed sequential loss of CD19 and CD22 expression after exposure to Blinatumomab and InO, respectively. At diagnosis, homogenous CD19 expression was present (**c**), which was lost at relapse after Blinatumomab-treatment (**d**). CD22 surface expression was documented before initiation of InO (**e**), but the patient rapidly developed a CD22-negative relapse (**f**). In patient 3, immunophenotyping revealed a CD22^dim^/CD34^bright^ subpopulation (21.3% of leukaemic cells) before initiation of salvage-therapy (**g**), which persisted after treatment with InO (**h**)

This 57-year-old male received the diagnosis of a Ph-positive common B-ALL in February 2014. Immunophenotyping revealed a common B-ALL phenotype with expression of CD45^dim^, CD34^+^, CD10^+^, CD19^+^ and CD22^+^ (Fig. 2a). Detailed molecular and cytogenetic analyses revealed *bcr/abl* rearrangement, trisomy 11, del12p13 and an amplification of 21q22. After induction treatment according to GMALL recommendations for elderly (> 55 years) patients which included imatinib, the patient showed persistent disease. Thus, treatment was switched to InO and the patient was enrolled into the INO-VATE trial (NCT01564784) and received a total of 3 cycles of InO [1]. A complete remission was achieved after cycle 1 and a complete molecular response (*bcr/abl* undetectable by PCR) was recorded after cycle 2. Subsequently, he received allogenic stem cell transplantation from an unrelated donor. The patient remained in remission for around 10 months, until he presented with a relapse in August 2015. Upon relapse, flow cytometric evaluation of the leukaemic cells revealed loss of CD22-expression (Fig. 2b). The patient was proceeded to best-supportive care.

### Patient 2

This 82-year-old female patient was diagnosed with Ph-negative common B-ALL in October 2014. A complete remission was achieved after induction treatment according to the GMALL treatment recommendations for elderly (> 55 years) patients. However, a first relapse occurred during consolidation chemotherapy in February 2015. The phenotype was CD45^dim^, CD34^+^, CD10^+^ and CD19^+^ (Fig. 2c). The patient received a total of 6 cycles with the bispecific T-cell engager anti-CD19/CD3-antibody Blinatumomab and achieved molecular complete remission after cycle 2. In January 2016, the patient presented with a CD19-negative relapse (Fig. 2c). Treatment with the tyrosine kinase inhibitor sorafenib resulted in a transient complete remission lasting 3 months. In April 2016, the patient developed her 3rd relapse. Flow cytometric evaluation of the leukaemic cells confirmed homogeneous expression of CD22 (Fig. 2e) and the patient was switched to InO. A complete remission was attained after cycle 2 and treatment was continued for a total of 4 cycles. Shortly after treatment discontinuation, the patient presented with her 4th relapse. Immunophenotyping revealed a loss of CD22 expression of the leukaemic cell population (Fig. 2f). With little further treatment options, the patient was proceeded to best-supportive care.

### Patient 3

This 24-year-old male was referred from an external hospital in February 2020 with relapsed, Ph-negative common B-ALL. Initially, this patient had been treated according to the GRAALL2003 protocol and attained a CR after first induction. However, he developed a relapse after the fourth consolidation chemotherapy cycle. At the time of relapse, the diagnostic work-up revealed expression of CD45^low^, CD10^+^, CD19^+^, CD20^−^, CD22^+^, CD34^+^ and TdT^+^. Two distinct populations were detected with varying expression of CD22 and CD34. While the majority of leukaemic cells exhibited CD22^+^ and CD34^+^, both with intermediate fluorescence intensity, a subpopulation (21.3% of leukaemic cells) showed expression of CD22^+(dim)^ and CD34^+(bright)^ (Fig. 2g). The patient received one cycle of InO monotherapy. Despite initial normalization of peripheral blood counts, bone marrow aspiration on day 26 was inconclusive (dry tap), but touch imprints showed submaximal marrow infiltration with leukaemic cells. Repeated immunophenotypic evaluation from peripheral blood detected 12% leukaemic cells. Interestingly, the phenotype was now homogeneous and identical to the previously detected minor subpopulation with dim fluorescence intensity for CD22 and bright fluorescence intensity for CD34 (Fig. 2h). The leukaemic cell population with intermediate fluorescence for CD22 and CD34 had vanished suggesting clonal selection under InO. After treatment failure was documented, the patient was switched to Blinatumomab. Unfortunately, the disease proved refractory to Blinatumomab, and the patient was released to best-supportive care. Repeated immunophenotyping revealed an unmodified immunophenotype with CD34^+(bright)^ and CD22^+(dim)^. However, CD19 expression was not lost after one cycle of Blinatumomab (data not shown).

### Comparative analysis

**Fig. 3:**
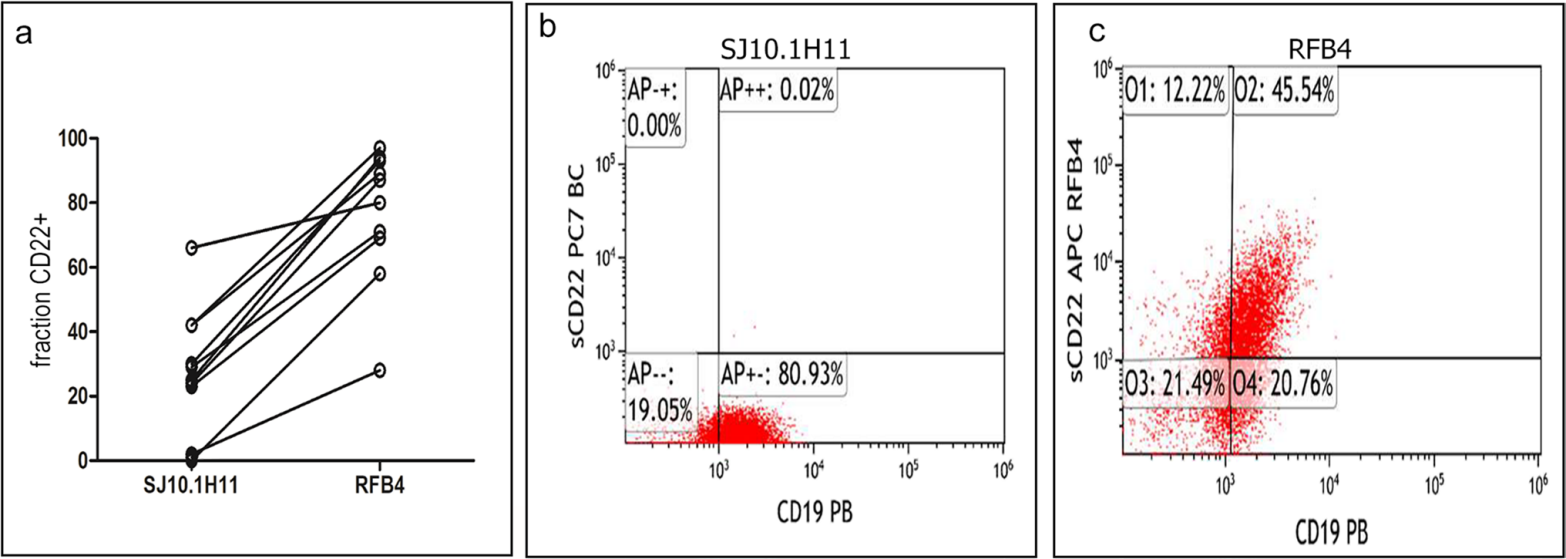
Comparative analysis of surface CD22-expression using the antibody clones SJ10.1H11 and RFB4 was performed in 10 cases of B-ALL (**a**). An example of a case is shown, in which CD22-expression was below the predefined, diagnostic threshold of 20% using SJ10.1H11 (**b**), but clearly above 20% using RFB4 (**c**)

For comparison of diagnostic antibody clones RFB4 and SJ10.1H11, we analysed 10 samples of patients with B-lineage ALL: 2 pro-B ALL, 7 common B-ALL and 1 pre-B-ALL. In all samples, a significantly higher proportion of CD22^+^ cells were detected among leukaemic cells with the use of the antibody clone RFB4 compared to SJ10.1H11 (median 84 versus 27, *p* < 0.01; Fig. 3a). In two samples, the predefined diagnostic threshold of 20% positivity was surpassed only by staining with the RFB4 antibody clone. Representative plots of one of these samples are shown in Fig. 3b, c.

## Discussion

Diagnosis and risk-stratification of lymphoblastic leukaemia rely on cytomorphology, cytogenetics, molecular genetics and immunophenotyping. It is advisable to repeat these analyses during relapse to guide the selection of appropriate salvage-therapies. Targeted therapies, such as InO, offer high efficacy and are well tolerated [3]. Therefore, the use of immunophenotyping to evaluate haematologic malignancies for the expression of antigens that are amenable to targeted immunotherapy is widely applied. A growing number of targetable antigens can be tested, and their expression should be considered as a prerequisite before treatment with targeted therapies. Furthermore, evidence is emerging that therapeutic effects of targeted therapy correlate with the intensity of expression for various targeted antigens [4, 5]. In order to investigate the expression levels of a therapeutic target, comparison to respective isotype controls and to internal controls should be employed [6]. The CD22 antigen is restricted to B-cells and has been described to be expressed in the vast majority of B-ALL, but the intensity of expression can vary significantly among individual patients [1, 7, 8]. Flow cytometric assessment of CD22 expression can be complicated by the fact that staining intensity will be significantly reduced if processing and analysis of the sample are delayed [9]. Furthermore, CD22 is generally considered to show rather low expression on the surface even in normal B-cells [10]. While treatment with InO should be omitted in the rare cases of CD22^negative^ B-ALL, the relevance of attenuated (i.e. dim) antigen expression remains uncertain. In the INO-VATE trial (NCT01564784), the inclusion of a small group of CD22^negative^ or CD22^low^ cases was reported. Three out of five of these patients showed a response to anti-CD22 therapy with InO [1]. The evaluation of CD22-expression was performed by flow cytometry at both local and central laboratories. The two central laboratories used the diagnostic antibody clone RFB4 and reported higher expression levels than local laboratories [11]. The exact expression levels, comparison to isotype controls or comparison of diagnostic antibody clones, have not been reported from the INO-VATE trial [1]. In our experiments, the anti-CD22 clone RFB4 was found to detect a higher fraction of CD22 + leukaemic cells compared to the clone SJ10.1H11.

The issue of CD22^dim^ or CD22^negative^ subpopulations, as exemplified in patient 3, remains largely unstudied and was not addressed by the authors of the INO-VATE trial [1]. There are, however, anecdotal reports of responses in patients presenting with very dim to negative CD22 expression [12]. CD22^negative^ B-ALL and the presence of CD22^negative^ subpopulations have been linked to KMT2A-rearranged B-ALL [12, 13]. However, this alteration was not reported in any of the presented cases. Loss of antigen expression has been described as resistance mechanism to various immunotherapies, e.g. Blinatumomab and Rituximab, for several B-cell neoplasms, but comprehensive studies on the frequency of CD22 loss after InO treatment in B-ALL are lacking [14]. However, the available literature suggests that a loss of the CD22 antigen occurs infrequently, i.e. only in few cases with resistance to or relapse after InO [15]. CD22 loss after treatment with InO has been first described in a paediatric patient with B-ALL [16]. Ryland et al. described a homozygous frameshift-mutation in the CD22 gene to be responsible for antigen-loss in one case of an adolescent patient with relapsed B-ALL that caused loss of antigen-expression as assessed by flow cytometry [17]. Similarly, frameshift-mutations in the CD19 gene have been described to complicate CD19-directed therapy [18]. Moreover, loss of antigen expression after targeted therapies may impact flow cytometric evaluation for minimal residual disease (MRD) in B-ALL [8]. Therefore, assessment of MRD, largely relying on expression of antigens that have served as targets of prior antigen-directed therapies, might produce false-negative results. In this context, Cherian et al. described an approach for flow cytometric MRD detection that can be employed after CD19-directed therapies [19].

In conclusion, the presented cases and data underscore the relevance of standardized flow cytometric evaluation of therapeutic targets prior to treatment initiation. Loss of CD22 expression and expansion of pre-existing CD22^dim^ or CD22^negative^ subpopulations obviously represent resistance mechanisms to InO. Currently, specific guidelines for assessment of CD22 expression by flow cytometry are not available. The presented data on different diagnostic antibodies highlight the need for standardization of flow cytometry protocols. Larger studies on antigen expression and clinical outcomes could improve clinical decision: it is currently unclear what cut-off values (20% or higher) should be employed to define eligibility for treatment with InO. Moreover, our data show that surpassing predefined cut-off values in flow cytometric evaluation of CD22-expression profoundly depends on the used diagnostic antibody clones. The data from patient 3 exemplify how pre-existing subpopulations can be monitored by flow cytometry. This also highlights the importance of careful evaluation of various combination plots, as the distinction between the predominant leukaemic cell population and other subpopulation was only visible by the varying CD34 expression. In acute leukaemia, subpopulations of malignant cells might carry intrinsic resistance to a specific therapy (such as InO) due to differences in expression of the respective target (such as CD22). It might be hypothesized that relapses could arise from even much smaller subpopulations displaying very dim or negative CD22-expression. Although correlative studies to prove this hypothesis are lacking, clinicians should be cautious in selecting specific antigen-directed therapies, if very dim or negative cell subpopulations are detected by flow cytometry.

## Materials and methods

Immunophenotypic analyses of three patients with B-ALL in whom loss of expression of CD22 occurred after treatment with InO were performed by local laboratories as described before [20]. In brief, peripheral blood or bone marrow specimens were analysed using standardized panels of various monoclonal antibodies and expression of CD22 was regarded as positive in cases with ≥ 20% CD22 + leukaemic cells as defined by comparison to respective isotype controls. Antibody clones for CD22 and instruments used were as follows: Clone HD239 (Beckman Coulter, Krefeld, Germany) and FACS Canto II (Beckton Dickinson, Heidelberg, Germany) for patient 1, clone SJ10.1H11 and Navios flow cytometer (both Beckman Coulter, Krefeld, Germany) for patient 2, and clone SJ10.1H11 and FC500 flow cytometer (both Beckman Coulter, Krefeld, Germany) for patient 3. Kaluza software (Beckman Coulter, Krefeld, Germany) was used to create combination plots of leukaemic cell population before and after treatment with InO. The leukaemic cell population was selected based on low side-scatter and reduced expression of CD45. Samples from 10 additional patients, which were sent to the central diagnostic laboratory for immunophenotyping within the framework of the German Multicenter Study Group for Adult ALL (GMALL) at the timepoint of diagnosis, were analysed for comparison using two different diagnostic CD22-specific antibody clones. SJ10.1H11 (Beckman Coulter, Krefeld, Germany) and RFB4 (Thermo Fischer Scientific, Dreieich, Germany) were simultaneously applied in a standardized panel using a Navios flow cytometer and Kaluza software (Beckman Coulter, Krefeld, Germany). Adobe Photoshop CC (Adobe, San José, CA, USA) was used for formatting of figures.

## Data Availability

The data presented in this study are available on request from the corresponding author. Raw data are not publicly available due to patient information connected with original data files.
